# Perceived neighborhood disadvantage and poor chronic health in Israel

**DOI:** 10.1186/s13584-025-00695-3

**Published:** 2025-05-27

**Authors:** Sharon Stein Merkin, Kathleen Abu-Saad

**Affiliations:** Gertner Institute of Epidemiology and Health Policy, Sheba Medical Center, Tel Hashomer, Israel

**Keywords:** Neighborhood disadvantage, Social determinants of health, Socioeconomic status, Disparities

## Abstract

**Background:**

Social disparities in health persist in Israel despite universal health care. Few studies have focused on the impact of neighborhood disadvantage on health in a representative sample of the Israeli population while accounting for multiple socioeconomic factors. The objective of this study was to assess the independent association between perceived neighborhood disadvantage and self-reported poor chronic health.

**Methods:**

Self-reported poor chronic health was defined as (1) reported not very good/poor health, and (2) having a chronic health/physical problem for > = 6 months disrupting daily life activities. Neighborhood disadvantage was based on self-reported measures of residential environment (scale of dissatisfaction with transportation, parks, cleanliness, waste removal, noise, pollution, safety, and walkability) and social problems (dissatisfaction related to neighbors, and neighbors interacting to improve the environment). High levels of neighborhood problems were defined as top 25th percentile of dissatisfaction scales. Logistic regression models included incremental adjustment for sex, age, ethnicity/religion, immigration status, peripheral region and then income, education and employment status.

**Results:**

A total of *n* = 7,020 participants with non-missing data were included. High levels of neighborhood environmental and social problems were independently associated with poor chronic health even after adjustment for sex, age, ethnicity/religion, immigration status, and peripheral region, and remained statistically significant after additionally adjusting for income, education, employment and lifestyle factors (odds ratio (OR) 1.5, 95% confidence interval (CI) 1.2–1.9 for environmental problems; OR 1.3, 95% CI 1.1–1.6 for social problems).

**Conclusions:**

Living in areas of perceived disadvantage conferred health risks beyond those related to ethnicity or socioeconomic status. These findings suggest that neighborhood-level factors contribute significantly to health disparities in Israel and should be included in national efforts to evaluate and minimize these health disparities. Future research is needed to also consider objective measures of neighborhood disadvantage, in order to determine the more salient neighborhood measures with respect to health outcomes and to effectively develop targeted interventions to reduce area-level health disparities.

## Background

The World Health Organization (WHO) defines the social determinants of health (SDoH) as “the conditions in which people are born, grow, work, live and age, and the wider set of forces and systems shaping the conditions of daily life.” [1] These factors are considered the drivers of health inequities as defined by “the avoidable differences in health status seen within and between countries.” [1] Indeed, global evidence has shown clear associations between social measures of disadvantage, including common domains related to finances, education, employment, health care access, neighborhood environments, social support, and increased health risk. [2].

In Israel, most of the research and policy initiatives related to SDoH inequities have focused on health differences by commonly selected SDoH factors, including ethnicity, education, immigration, religiosity and degree of geographic peripherality. [3–7] Studies have generally found increased health risk among Arab Israelis compared to Jewish Israelis [4–7] and worse health profiles among immigrants from the former Soviet Union and elsewhere compared to those born in Israel. [7, 8] There is some conflicting research when comparing Ultra-Orthodox (known as “Haredi”) Jews to secular or religious non-Haredi Jews, with data indicating negative health behavior profiles among Haredim [9, 10] and other studies showing the opposite trend of better self-reported health and lower mortality levels in this group. [11, 12] Although negative health effects of living in peripheral areas of the country that are far from the center (and thus may have suboptimal access to health care resources) have been an important focus of Israeli health policy and research, [7, 13, 14] few studies have examined the association between measures of neighborhood disadvantage and health. [5, 11, 15].

Living in disadvantaged neighborhoods has been linked to increased risk of multiple negative health outcomes including diabetes, smoking, high body-mass index (BMI), high blood pressure, [16–19] cardiovascular disease [17, 20] and mortality. [21] Several pathways have been suggested to explain how living in disadvantaged neighborhoods may affect health, including limited access to recreational facilities, [22, 23] healthy and affordable food, [24–26] inadequate health care, [27] and increased exposure to violence and stressful life events. [28] These factors and others can operate individually and synergistically, via negative health behaviors or directly, to increase risk of disease.

Neighborhood disadvantage has been defined in research studies in myriad ways, most commonly using objective measures of residential areas based on compositional measures of socioeconomic indicators of residents per area (for example, percent of people living in poverty). [16–20] Other studies have considered subjective measures of neighborhood conditions reported by residents. [29–32] These subjective measures are insightful in a number of ways, for example, by including indicators that are contextual in nature (describing neighborhood infrastructure and resources) that are often lacking in Census-based measures, and can thus directly address the more proximate causes of health risks, such as lack of recreational spaces. [33] Moreover, some researchers suggest that the very perception of these characteristics, as opposed to an objective measure of socioeconomic status (SES), is the more influential factor on health. [34] Indeed, there is evidence that perceptions of neighborhoods have independent effects on health even after adjusting for objective measures of neighborhood poverty. [29, 33].

We hypothesize that living in perceived disadvantaged neighborhoods in Israel poses an additional health risk beyond the other social determinants previously considered. The objective of this study is to examine the distinct health impact of living in perceived disadvantaged areas even after accounting for ethnicity, immigration and individual-level SES, utilizing a representative sample of the Israeli population.

## Methods

### Study sample and design

The study sample is based on the Israel Central Bureau of Statistics (ICBS) Social Survey of 2017. The ICBS conducts a social survey annually since 2002 to provide updated information on welfare and living conditions of the Israeli population. These surveys are based on a national sample of the Israeli population, ages 20 and older (excluding prison inmates, residents of therapeutic institutions and unrecognized Bedouin villages), and include modules on housing, employment, general health, and financial status, as well as a module that focuses on a different topic each year. The last completed Social Survey with a focus on health was conducted in 2017 and utilized in the current study. The sampling frame was based on the Population Register file; large localities (> 8,300 residents ages > = 20), representing 83% of the population, were randomly sampled within 86 strata based on population ethnic, gender, age, religious, immigrant and education groups. Small localities were sampled in 2 stages, first by geographic size and then by the same population strata. [35] The total survey sample included *n* = 7,230 participants, with a 75% response rate. The survey was conducted via in-home interviews or by telephone, and average length of interview was approximately 30 min. [35] The analytic sample for this study included 7,020 of the total sample (97%) not missing data on the main variables of interest; *n* = 98 were excluded for missing perceived neighborhood measures, *n* = 24 for missing self-reported health, and *n* = 88 for missing socioeconomic measures.

#### **Exposure**: perceived neighborhood disadvantage

Participants were asked about their satisfaction with various aspects of their neighborhood on a Likert scale ranging from 1 (very satisfied) to 4 (not at all satisfied). Neighborhood disadvantage scores were created by summing these values and reverse coding when relevant so that higher scores indicate higher disadvantage. Two subscales were calculated using available neighborhood perception measures in the survey: neighborhood environmental problems (dissatisfaction with transportation, parks, cleanliness, waste removal, noise, pollution, safety and walkability; total range for score 1–32) and social problems (dissatisfaction with neighbors in general and with neighbor interaction to improve the environment; total range for score 1–8). While the types of social problems were limited in the survey, the environmental problems included similar items used in other studies and the scores were calculated in a similar way. [29, 32] The final measure of neighborhood disadvantage was defined as the top quartiles for each of these subscales (≥ 21 for environmental problems and ≥ 6 for social problems); the phi coefficient between these two binary measures was 0.20.

#### **Outcome**: self-reported poor chronic health

Self-reported poor chronic health was defined by combining responses to three questions about health where individuals reported “not very good” or “poor” health when asked about their general health status, and also reported having a chronic health or physical condition for at least 6 months that interferes with daily life activities (“interferes a lot” or “interferes”). While research has shown that poor self-reported health is a robust measure of poor health [36] encompassing multiple health domains, [37] we included the additional information asked of participants about chronic health burden that affects their daily life in order to more specifically assess self-reported poor chronic health. The phi coefficient between poor self-reported health and disruptive chronic health burden is 0.93. General poor self-rated health was considered as an alternate outcome in sensitivity analyses.

#### Covariates

We considered sociodemographic variables as potential confounders in the association between neighborhood disadvantage and poor chronic health. Demographic indicators included age (reported in 5-year age categories from 20 to 64, 65–74, >=75) and sex. Ethnicity was classified into major population groups in Israel that combine nationality and religiosity, as these groups have been identified as experiencing health disparities in other research, [5, 9] including Arab, Jewish Haredi (Ultra-Orthodox), and Jewish non-Haredi/other (reference; other includes those who identify as Jewish but do not report religious affiliation). Immigrant groups have also been identified in Israel as experiencing higher health risks than the native-born population, specifically those who emigrated from the former Soviet Union to Israel in and after 1990. [11] The Social Survey therefore asks specifically about immigration from the former Soviet Union and otherwise reports birthplace based on continents. Immigrant groups in this study were thus classified into the following categories of regions of birth: former Soviet Union, America/Europe, Asia, Africa (reference: born in Israel). Living in peripheral regions of the country was classified as North, South, West Bank vs. Center (including Jerusalem, Tel Aviv, Haifa and other central areas). Income per capita was derived from reports of household income and number of household members and categorized as < = 2,000 NIS, 2,001–4,000 NIS vs. >4,000 NIS (an additional category was included for missing income considering the relatively high number of missing values; see Table 1). Ability to cover monthly household expenses related to food and utilities was based on participant responses of “not at all able” or “not so able” (vs. “able without difficulty” or “able”). Education was based on highest attained degree or certificate and classified as: never attended education institution, no attained degree, completed high school without matriculation certificate (a compulsory requirement for higher education [38]), completed high school with a matriculation certificate, vocational degree and Bachelor’s or higher degree (reference). Employment status was classified as currently employed vs. unemployed. Lifestyle factors included smoking (current, past vs. never), BMI (obesity defined as BMI > = 30.0; overweight as BMI between 25.0-29.9) [39] and a recommended level of physical activity, defined based on reporting at least 150 min of moderate and/or strenuous physical activity. [39].

**Table 1:** Select characteristics of social survey 2017 Participants, n = 7,020

	%
Neighborhood Perception	
High Neighborhood Environmental Problems	26.4
High Neighborhood Social Problems	24.1
**Sex**	
Female	50.7
Male	49.3
**Age**	
20–34	31.2
34–49	28.5
50–64	21.3
65+	18.9
**Ethnic Group**	
Arab	19.0
Jewish Haredi	8.1
Jewish (non-Haredi)/Other	72.9
**Immigration (place of birth)**	
Former Soviet Union	14.8
America/Europe	7.4
Asia	3.1
Africa	5.2
Israel	69.6
Peripheral Region*	
North	17.6
South	12.3
West Bank	3.5
Center	66.6
**Income per capita**	
<=2,000 NIS	17.0
2,001–4,000 NIS	22.7
> 4,000 NIS (Reference)	38.5
Missing	21.8
**Difficulty/Unable to cover expenses**	30.8
**Education (highest degree)**	
Never attended educational institution	1.2
No attained degree	15.3
Complete HS no matriculation certificate	15.0
Complete HS + matriculation certificate	19.8
Vocational Certification	16.4
BA/First or Higher degree	32.4
**Employment**	
Currently Employed	69.0
Unemployed	31.0
**Smoking**	
Current	21.9
Past	18.4
Never	59.7
**BMI**	
Obese (BMI > = 30.0)	15.9
Overweight (BMI 25.0-29.9)	33.3
Normal (BMI < 25.0)	44.8
Missing	6.0
Recommended Physical Activity**	26.9
Self-Reported Poor Chronic Health***	9.6
Self-Reported Poor Health	10.9
Chronic Health Burden	33.4
Chronic Health Burden Interfering with Daily Life	21.6

*Participants in the West Bank include only Jewish Israelis; Center region encompasses Jerusalem, Haifa, Tel Aviv and other central areas

**at least 150 min of moderate and/or strenuous physical activity per week

*** Main Outcome: Composite variable that includes those who report poor health (“not very good” or “poor”), plus a chronic health problem lasting > = 6 months that interferes with daily life

### Statistical analysis

Distributions of all variables of interest were assessed overall and by self-reported poor chronic health. Initial logistic regression models were used to assess the association between neighborhood disadvantage and self-reported poor chronic health adjusted for age, sex, ethnicity/religion, immigration, and peripheral region. Both variables for high neighborhood environmental problems and high neighborhood social problems were considered in the same model. In order to assess the role of individual SES in mediating this relationship, a second set of models additionally adjusted for income per capita, inability to cover expenses, education and employment. A third set of models further adjusted for lifestyle factors (smoking, BMI levels and physical activity) to determine if these factors explained observed disparities by neighborhood disadvantage. Interaction terms between neighborhood problems and sex and ethnic/religious groups were tested in the fully adjusted model to assess if the relationship between perceive neighborhood conditions and health differs by these groups. In sensitivity analyses, we repeated models for the more general outcome of poor self-reported health. All analyses were conducted using SAS version 9.4. [40].

## Results

Distributions of all the variables of interest (Table 1) indicated that about 60% of the study sample was aged < 50 years, 19% were Arab Israeli, 8% were Haredi, 15% were immigrants from the former Soviet Union. 33% lived in peripheral regions of the country, 31% reported having difficulty or unable to cover their monthly expenses, 32% never obtained an educational degree or a high school matriculation certificate, and 31% are unemployed. These distributions are similar to the original survey sample before exclusions (data not shown). In addition, 16% of the sample reported high BMI levels ( > = 30), and 27% reported engaging in recommended levels of physical activity. A total of 10% of the sample reported poor levels of chronic health.

The initial logistic regression model, adjusted only for sex and age, showed that those living in disadvantaged neighborhoods had a little over twice the risk of self-reported poor chronic health when defined by environmental conditions (odds ratio (OR) 2.1, 95% confidence intervals (CI) 1.7–2.5) and 70% higher risk when defined by social problems (OR: 1.7, 95% CI: 1.4-2.0). This risk was mitigated somewhat when adjusting for ethnicity/religion, immigration, and living in a peripheral region, but remained statistically significant (OR: 1.6, 95% CI: 1.3-2.0 for environmental problems and OR: 1.5, 95% CI: 1.3–1.9 for social problems). This association between neighborhood disadvantage and poor chronic health was slightly reduced but remained statistically significant after additional adjustment for individual-level SES (OR: 1.5, 95% CI: 1.2–1.9 for environmental problems and OR: 1.3, 95% CI: 1.1–1.6 for social problems), and remained unchanged after additional adjustment for lifestyle factors in the final model (all models are listed in Table 2). Interaction terms for neighborhood measures and sex yielded no statistical significance. Interaction terms for neighborhood measures and ethnicity/religion were significant only for neighborhood social problems and Haredi identity (*p* = 0.04); stratified models showed much stronger association between neighborhood social problems among Haredi participants (OR 13.4 95%CI 1.6-115.5; data not shown) compared to non-Haredi participants (similar to main results), although confidence intervals are wide. Overall results were similar in sensitivity analyses considering poor self-rated health as the outcome, with a slightly increased OR for neighborhood social problems in the fully adjusted model (OR: 1.4, 95% CI 1.2–1.8; data not shown).

**Table 2:** Odds ratio of self-reported poor chronic health by neighborhood disadvantage

	Model 1*	Model 2*	Model 3*	Model 4*
Perceived Neighborhood Disadvantage	Odds Ratio (95% Confidence Interval)			
High (vs. Low) Neighborhood Environmental Problems	**2.1 (1.7**,** 2.5)**	**1.6 (1.3**,** 2.0)**	**1.5 (1.2**,** 1.9)**	**1.5 (1.2**,** 1.9)**
High (vs. Low) Neighborhood Social Problems	**1.7 (1.4**,** 2.0)**	**1.5 (1.3**,** 1.9)**	**1.3 (1.1**,** 1.6)**	**1.3 (1.1**,** 1.6)**
**Ethnic/Religious Group**				
Arab		**4.4 (3.3**,** 5.7)**	**1.6 (1.2**,** 2.2)**	**1.4 (1.0**,** 1.9)**
Jewish Haredi		0.9 (0.5, 1.4)	**0.5 (0.3**,** 0.8)**	**0.4 (0.3**,** 0.8)**
Jewish (non-Haredi)/Other		Reference	Reference	Reference
**Immigration (place of birth)**				
Former Soviet Union		**2.1 (1.6**,** 2.7)**	**2.4 (1.8**,** 3.2)**	**2.2 (1.7**,** 3.0)**
America/Europe		0.9 (0.6, 1.3)	1.0 (0.7, 1.4)	0.9 (0.6, 1.4)
Asia		1.5 (1.0, 2.2)	1.1 (0.7, 1.7)	1.0 (0.8, 1.6)
Africa		**1.9 (1.4**,** 2.7)**	1.4 (1.0, 2.0)	1.3 (0.9, 1.9)
Israel		Reference	Reference	Reference
Peripheral Region**				
North		1.0 (0.8, 1.3)	1.0 (0.7, 1.2)	1.0 (0.7, 1.3)
South		**1.6 (1.3**,** 2.1)**	**1.5 (1.1**,** 1.9)**	**1.5 (1.1**,** 2.0)**
West Bank		1.4 (0.7, 2.5)	1.4 (0.7, 2.6)	1.2 (0.6, 2.3)
Center		Reference	Reference	Reference
**Income (monthly) per capita**				
<=2,000 NIS			1.3 (1.0, 1.8)	1.3 (0.9, 1.8)
2,001–4,000 NIS			**1.4 (1.1**,** 1.8)**	**1.4 (1.1**,** 1.8)**
> 4,000 NIS (Reference)			Reference	Reference
Missing			1.0 (0.7, 1.3)	0.9 (0.7, 1.2)
**Difficulty covering expenses**			**2.1 (1.7**,** 2.6)**	**2.0 (1.6**,** 2.5)**
**Education (highest degree)**				
Never attended educational institution			**4.1 (2.3**,** 7.4)**	**3.0 (1.7**,** 5.5)**
No attained degree			**2.2 (1.6**,** 3.0)**	**1.8 (1.3**,** 2.4)**
Complete HS no matriculation certificate			**1.9 (1.4**,** 2.7)**	**1.7 (1.2**,** 2.3)**
Complete HS + matriculation certificate			1.0 (0.7, 1.5)	0.9 (0.6, 1.3)
Vocational School			1.3 (0.9, 1.7)	1.3 (0.9, 1.7)
BA/First or Higher degree			Reference	Reference
**Employment**				
Currently Employed			**0.3 (0.2**,** 0.3)**	**0.2 (0.2**,** 0.3)**
Unemployed			Reference	Reference
**Smoking**				
Current				**1.3 (1.0**,** 1.7)**
Past				1.2 (1.0, 1.6)
Never				Reference
**BMI**				
Obese				**1.9 (1.4**,** 2.4)**
Overweight				1.0 (0.8, 1.3)
Normal				Reference
Missing				**1.5 (1.0**,** 2.1)**
Recommended Physical Activity***				**0.4 (0.3**,** 0.5)**

Bold results indicate statistical significance, *p* < 0.05

*Incrementally adjusted models; all models adjusted for sex and 5-year age categories from 20–64, 65–74, >=75; OR: Odds Ratio; CI: confidence interval; BMI: Body-Mass-Index. Bold indicates *p* < 0.05

** Participants in the West Bank include only Jewish Israelis; Center region encompasses Jerusalem, Haifa, Tel Aviv and other central areas

***at least 150 min of moderate and/or strenuous physical activity per week

Other measures of SDoH indicated statistically significant associations with poor chronic health, after adjustment for age, sex and neighborhood disadvantage. Interestingly, most of these associations remained stable and mostly unchanged even after adjustment for other factors. Participants who emigrated from the former Soviet Union had over twice the risk of poor chronic health (OR: 2.2, 95% CI: 1.7-3.0 in fully adjusted model). Living in the southern part of the country was associated with higher levels of poor health that remained independent of neighborhood, individual SES or other demographic or lifestyle factors (OR: 1.5, 95%CI: 1.1-2.0). Difficulty covering expenses, lower income levels and unemployment were also independently associated with higher levels of poor chronic health, as was no educational degree or less than completed high school matriculation (see Table 2). The most striking measure of health disparities explained by some of these covariates was ethnicity/religion, with Arab ethnicity associated with over four times the risk of self-reported poor chronic health after adjusting for peripheral region, neighborhood disadvantage, age and sex; after additional adjustment for individual-level SES, this association was reduced to OR 1.6, 95% CI: 1.2–2.2 and slightly further with lifestyle factors. With regard to lifestyle factors, current smoking levels, obesity and less than recommended levels of physical activity were all associated with self-reported poor chronic health.

## Discussion

Our findings indicate that neighborhood environmental and social conditions function as SDoH, independent of other socioeconomic and social factors. Specifically, we found that individuals living in areas they describe as disadvantaged experience increased self-reported poor chronic health, even after accounting for ethnic/religious, immigration and socioeconomic status; this association is apparent for both neighborhood environmental physical conditions and social problems.

While our study confirms other research demonstrating persisting health disparities in Israel despite universal health care for all citizens, the current analyses show the importance of considering neighborhood factors as an additional social determinant of health that has been neglected in Israel Ministry of Health (IMOH) reports of inequality. [41] Our findings of an independent association between neighborhood conditions and health, even after accounting for other commonly considered SDoH, indicate that this is another important domain to consider when evaluating and addressing national health inequities. These findings confirm similar results found in another Israeli study on self-reported health, [5] highlighting the importance of considering multiple SDoH to fully address population health disparities.

These results also indicate the importance of considering various aspects of neighborhood measures to better understand population disparities in health in Israel. In most studies of perceptions of neighborhood conditions, dimensions of physical and social environments are considered separately, [30–34] reflecting distinct pathways linking neighborhood conditions to a variety of health outcomes. [42] Our results indeed show that social and environmental dimensions of neighborhood perceptions independently influence health, although the magnitude of this association is greater for the latter. Similar to our results, other studies also found physical/environmental neighborhood problems to be more strongly associated compared to social problems, [30, 32, 34] although the difference in our analyses may also be due to relatively fewer measure of neighborhood social concerns. Interestingly, among the minority Haredi population, we found a stronger association between neighborhood social problems and poor health compared to the association in the non-Haredi population. While this sub-sample was too small to provide conclusive evidence, these findings support research showing the particularly strong relationship between sense of community, social ties and health among the Haredi population. [43] On a broader level, these findings suggest that differing aspects of neighborhood perceptions may influence health differently in different cultural/ethnic groups. Results of the current study also provide further evidence of the importance of considering measures of disadvantage at small residential areas when available (rather than city-level indicators) so as to better describe living environments, as well as the importance of considering neighborhood measures as independent indicators of risk and not merely proxies of individual-level SES. [44, 45].

Indeed while the main aim of this study was to assess the relationship between neighborhood disadvantage and health, our findings additionally confirm the independent associations between some of the other domains of SDoH. Specifically, in this Israeli population-based sample we found higher levels of self-reported poor chronic health among those of Arab ethnicity (compared to non-Haredi Jews and others), immigrants from the former Soviet Union (compared to those born in Israel), those living in the southern region of Israel (compared to the center), those experiencing difficulty or unable to cover their monthly expenses, those with minimal or no education, and those unemployed (Table 2). In incremental models, certain factors explained some of the observed disparities (for example, Arab-Jewish differences were significantly reduced after adjusting for individual-level SES). All these separate factors remained significantly associated with poor chronic health in the fully adjusted model and reflect multiple and intersecting domains of SDoH that can be targeted for intervention.

### Health policy implications

Health policy, in considering the contributions of several domains of the SDoH that (as our analyses indicate) operate independently and synergistically, must determine how best to target vulnerable populations to mitigate and prevent morbidity and mortality and to narrow health disparities. Neighborhood-level disadvantage has not been considered as an independent SDoH domain with regard to health policy in Israel, as reflected in recently published guidelines establishing a set of national health equity indicators that do not distinguish between neighborhood and individual-level socioeconomic conditions. [46] Our current findings suggest two main reasons for directly targeting neighborhood-level factors in an effort to improve population health and minimize health disparities, including (1) neighborhood/area-level factors may directly impact health and (2) targeting neighborhoods or areas rather than individuals may be a more cost-effective and efficient approach to affect change.

Our study confirms in Israel what many other researchers have found globally, that neighborhood disadvantage negatively impacts health independent of individual-level measures of disadvantage. [16, 17, 20, 21, 29] This persistent finding suggests that directly targeting area-level infrastructure may then influence the health of individuals living in those areas. Examples of such policies may include improving transportation to allow for access to resources outside that area; building recreational spaces to encourage physical activity; promoting the affordability and accessibility of healthy foods. [47] Recent reviews have indicated that interventions targeting neighborhood-level physical [48, 49] (e.g., green space, walkability, health/unhealthy food outlets) and social [50–52] (e.g., crime, disorder) factors may improve health behaviors and physical and mental health outcomes. However, the evidence of health impact from such programs remains inconclusive, [53–56] as does the generalizability of interventions across different countries, cultures and societies. Thus, health policy measures in Israel should consider the unique factors related to the local population; these may be ascertained through within-locality focus groups and stakeholders to determine neighborhood conditions that are most in need of improvement.

Indeed, the IMOH’s most recent systemic program to reduce Arab-Jewish health disparities included an example of such an effort by mapping the conditions of health-promoting infrastructure (for example, sidewalks, street lights and parks) in Druze and Circassian towns, and initiating a multi-departmental, multi-level government initiative to improve this local physical infrastructure. [41] Much of the funding to support this project comes from the 2015 Government Resolution 922 (GR-922) Five Year Economic Development Plan for Arab Society (and carried over to subsequent 5-year plans), enabling the replication of such projects in other Arab towns with suboptimal infrastructure. [41, 57] These efforts have resulted in successful pilot projects in some Arab towns (for example, an affordable housing complex was built in Sakhnin in the Upper Galilee, that includes wide sidewalks to encourage pedestrian activity). However, the State Comptroller reported that the actual implementation of such projects has been very low, and most of the funding remains under-utilized, at least partially due to unrealistic projects or lack of awareness of the availability of new resources. [57] Our findings highlight the important policy potential of mapping health-promoting infrastructure needs at the neighborhood level in communities in peripheral regions to support the planning, monitoring and evaluation of such neighborhood-level interventions and to ultimately reduce health disparities. Moreover, these policy recommendations require cooperation across different government agencies, as reflected by the various types of neighborhood complaints measured in the current study that impact health, including safety, effective public transportation, walkability, recreational spaces and cleanliness.

Our second stated reason for supporting policy efforts to target area-level factors in order to impact individuals’ health involves the practicality and cost-effectiveness of such an approach. That is, even if neighborhood-level and health associations were ultimately driven by individual-level socioeconomic and other social/ethnic disparities that are not measured (i.e. a situation where area-level is only a proxy for individual-level SDoH), area-level interventions may still be the more effective way to impact health for a number of reasons. First, individual-level SDoH are often difficult or impossible to modify directly (i.e. ethnicity/religiosity, income levels), whereas improving area-level infrastructure and planning is already within the strategic objectives of government agencies devoted to those efforts. Second, aside from providing physical resources for health-promoting behaviors, there is evidence that community-level interventions and policies influence individuals through a social-ecologic model of behavior change where the environment is a source of encouragement and support for positive change in health behavior. [58] Finally, targeting area-level change to improve both areas of residence as well as the health of residents, is a “win-win” approach to improve the health of individuals as well as the context in which they live (the very definition of SDoH). Improving these conditions will promote positive health behaviors and ensure positive conditions to maintain these improvements.

### Strengths and limitations

The strength of this study includes the diversity of the study sample, representative of the Israeli population and including distinct ethnic and religious minority groups. Despite this strength, there are some limitations to note, including the time period of the survey, conducted in 2017. The ICBS conducts the Social Survey annually, however, includes health-related questions only every several years; data from the most recent survey including health data are not yet available. Additional limitations of this study include the self-reported health measure as well as the self-reported measures of neighborhood disadvantage based on participant survey responses. While further research should include objective measures of health as well as neighborhood markers, there is research that suggests that self-reported health is a powerful indication of overall wellbeing [36] and that perception of neighborhood environment may directly influence health. [34] Nonetheless, these points highlight the need for future research to examine the association between objective measures of neighborhood conditions as well as biological and medical measures of health.

## Conclusions

Living in areas of perceived disadvantage conferred health risks beyond those related to other SDoH. These findings highlight the need for collecting additional neighborhood-based measures of environment, infrastructure and resources to address health disparities and to inform policy and implement interventions.

## Data Availability

The datasets analyzed in the current study are available upon request from the Israel Central Bureau of Statistics: https://www.cbs.gov.il/en/subjects/Pages/Social-Survey.aspx.

## Abbreviations

OR: Odds ratio
CI: Confidence interval
WHO: World Health Organization
SDoH: Social determinants of health
BMI: Body-mass index
SES: Socioeconomic status
ICBS: Israel Central Bureau of Statistics
IMOH: Israel Ministry of Health
